# Ubiquitin-Specific Peptidase 7: A Novel Deubiquitinase That Regulates Protein Homeostasis and Cancers

**DOI:** 10.3389/fonc.2021.784672

**Published:** 2021-11-19

**Authors:** Lin Zhou, Taohui Ouyang, Meihua Li, Tao Hong, Alriashy MHS, Wei Meng, Na Zhang

**Affiliations:** ^1^ First Clinical Medical College, Nanchang University, Nanchang, China; ^2^ Department of Neurosurgery, The First Affiliated Hospital of Nanchang University, Nanchang, China; ^3^ Department of Neurosurgery, Huashan Hospital of Fudan University, Shanghai, China; ^4^ Department of Neurology, The First Affiliated Hospital of Nanchang University, Nanchang, China

**Keywords:** ubiquitin-specific peptidase 7, deubiquitinase, cancer, neoplasms, protein homeostasis, proteostasis

## Abstract

Ubiquitin-Specific Peptidase 7 (USP7), or herpes virus-associated protease (HAUSP), is the largest family of the deubiquitinating enzymes (DUBs). Recent studies have shown that USP7 plays a vital role in regulating various physiological and pathological processes. Dysregulation of these processes mediated by USP7 may contribute to many diseases, such as cancers. Moreover, USP7 with aberrant expression levels and abnormal activity are found in cancers. Therefore, given the association between USP7 and cancers, targeting USP7 could be considered as an attractive and potential therapeutic approach in cancer treatment. This review describes the functions of USP7 and the regulatory mechanisms of its expression and activity, aiming to emphasize the necessity of research on USP7, and provide a better understanding of USP7-related biological processes and cancer.

## 1 Introduction

**Figure 1:**
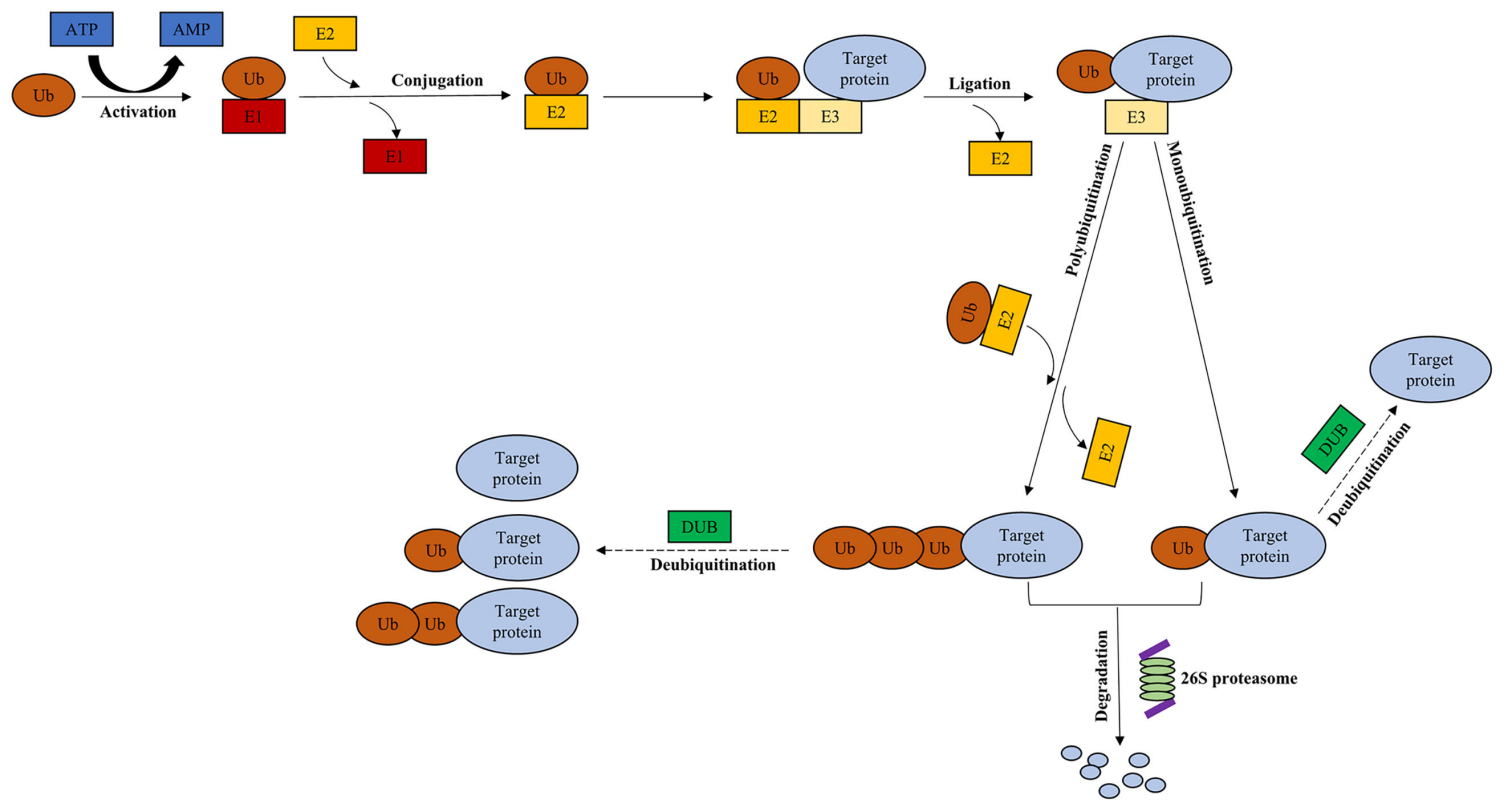
Ubiquitin-proteasome system. Ubiquitination of the target protein is catalyzed through E1, E2, and E3. Ub is activated by E1 in an ATP-dependent manner, which is subsequently transferred to E2. Then, an isopeptide bond is formed following the conjugation between ubiquitin and a lysine residue of the target protein *via* E3. Finally, the target protein is recognized by 26S proteasome, leading to the degradation of protein. The presence of DUBs rescues the target protein from being degraded, and contributes to its stabilization through deubiquitination. Dotted arrow: deubiquitination. Ub, ubiquitin; E1, ubiquitin‐activating enzyme; E2, ubiquitin‐conjugating enzyme; E3, ubiquitin ligase enzyme; DUB, deubiquitinating enzyme.

The ubiquitin-proteasome system (UPS), comprising ubiquitin, 26S proteasome, and four families of enzymes [ubiquitin‐activating enzyme E1, ubiquitin‐conjugating enzyme E2, ubiquitin ligase enzyme E3, and deubiquitinating enzymes (DUBs)] (1), regulates most intracellular protein degradation, cellular functions and maintains protein homeostasis (2) (**Figure 1**). The UPS is involved in many biological processes, including immune response, cell cycle progression, signal transduction, and tumorigenesis (3). Ubiquitination, one of the most important posttranslational modifications (PTMs), takes part in regulating the stability, localization, and activity of proteins (4). The balance between protein synthesis and degradation is essential for homeostasis in cells (5). Ubiquitin is conjugated to the target protein *via* an enzymatic cascade, including E1, E2, and E3 (1). First, E1 activates ubiquitin, which is mediated by ATP (4). Subsequently, ubiquitin is transferred to E2 through trans-thiolation (4). Then, an isopeptide bond is formed following the conjugation between ubiquitin and a lysine residue of the target protein (6), subsequently recognized by 26S proteasome (5). Finally, the isopeptide bond is hydrolyzed by deubiquitinating enzymes (1).

As one of the parts within UPS, the functions of DUBs are to reverse and antagonize the ubiquitination effect on the target protein, and participate in the balance between ubiquitination and deubiquitination. Recent studies have shown that, the aberrant activity and expression levels of DUBs correlates to numerous diseases, such as cancer.

DUBs mainly consist of 7 families: ubiquitin-specific proteases (USPs) (the largest family), ovarian tumor proteases (OTUs), Ub C-terminal hydrolases (UCHs), Machado–Joseph disease protein domain (MJD) proteases (MJDs), Jab1/MPN domain-associated metallo-isopeptidases (JAMM/MPM+), monocyte chemotactic protein-induced proteases (MCPIPs), and Zinc finger UB-specific proteases (ZUP/ZUFSP) (4).

Among the USPs family, ubiquitin-specific protease 7 (USP7), or herpes virus-associated protease (HAUSP), is well- characterized. USP7 is a multi-domain protein that contains 1102 amino-acid residues (5), including (i) a catalytic domain (CD) with finger, thumb, and palm domain (7), (ii) a C-terminal domain that contains ubiquitin like domains (UBL) 1-5 (4), and (iii) a tumor necrosis factor (TNF) receptor associated factors (TRAFs) like domain in its N-terminal domain (4). The catalytic domain participates in cleaving the peptide bond between the substrates of USP7 and ubiquitin (8). Unlike other USPs, the conserved catalytic domain of USP7 has no catalytic capacity (4), which makes USP7 quite unique compared with other USPs. Both of the UBL domains in the C-terminal domain and the TRAFs like domain in the N-terminal domain are able to recognize multiple substrates of USP7 (8). The TRAFs like domain has been proved to interact with p53 and MDM2/MDMX (1, 9). A recent study also shows that, deleting the TRAF-domain effects nuclear localization of USP7 (4). Different UBL domains recognize different substrates: UBL1-2 has the binding sites of ring finger protein (RNF) 168 and 169 (the negative regulator of RNF168), xeroderma pigmentosum complementation group C (XPC), DNA methyltransferases 1 (DNMT1), and ICP0 (10–14). HDM2 is recognized by UBL3 (8, 15), and UBL4-5 binds the tumor suppressor p53 (8, 15) and forkhead box O4 (FOXO4) (8, 16). In addition, the C-terminal domain also plays a key role in modulating the catalytic activity of USP7 through regulating the activity or localization of the enzyme (17), the incomplete structure of which greatly impairs USP7 activity (15, 18).

USP7 functions as an essential role in different cell processes through interacting with its multiple substrates (**Table 1**), such as DNA damage and repair, immune responses, epigenetic control, and tumor progression (4). The expression and activity of USP7 are also regulated by multiple regulators through gene transcription and PTMs. Recently, many studies have shown that dysregulated cell processes mediated by USP7 may contribute to numerous diseases, such as cancers. Moreover, USP7 with aberrant expression levels and abnormal activity also correlates with cancers. Therefore, given the association between USP7 and cancers, targeting USP7 could provide a potential therapeutic strategy in cancer treatment.

**Table 1:** Substrates or related proteins of USP7.

Substrates or related proteins	Processes	References
NF-κB	Inflammatory signaling	(19)
HSCARG	NF-κB signaling	(20, 21)
IKK-γ^a^ TRAF6^b^	Innate immunity	(22)
ICP0	Innate immune response Viral infection	(22, 23)
LANA^c^	Viral infection	(24)
vIRF^d^		(25, 26)
RTA^e^		(27)
E1B‐55 K		(28)
UL35		(29)
EBNA1^f^		(30)
Foxp3^g^	Tumor evasion	(31)
VP24 protein	Innate immune evasion	(32)
Tip60	DNA damage responses Cell apoptosis Tumor evasion	(21, 31, 33, 34)
P53	DNA damage repair DNA damage responses Cell proliferation, migration, and apoptosis Tumor progression Tumor suppression	(1, 8, 31, 35–40)
MDM2	P53 degradation Cell proliferation Cell apoptosis	(31)
HDM2, HDMX	P53 destabilization and downregulation	(8)
CCDC6^h^	DNA damage responses DNA repair	(1)
CHK1^i^	Double-strand DNA breaks Cell cycle Tumor resistance	(1, 8, 41–43)
XPC^j^	Nucleotide excision repair (NER)	(10)
CSB^k^/ERCC6	Transcription‐coupled nucleotide excision repair (TC‐NER)	(1, 44, 45)
HLTF^l^	DNA damage bypass Damaged gene replication Cell cycle	(1, 46)
CHFR^m^	Cell cycle	(47)
Cyclin A2		(48)
Ki-67 antigen		(49)
UHRF1^n^	Replication	(1, 50)
Histone LSD1	Cell proliferation and migration	(4)
MDC1°	Double-strand DNA breaks Homologous recombination	(51)
PHF8^p^	DNA damage responses Cell cycle	(1, 4, 48, 52)
ANXA1^q^	DNA damage responses Cell apoptosis	(53)
Rad18	DNA damage tolerance Cell cycle	(46)
RNF168^r^ RNF169^s^	DNA damage responses	(4, 8, 11, 12)
DNA polymerase polη	DNA damage	(54, 55)
RNA polymerase II	CSB stabilization Nucleotide excision repair (NER)	(1)
UVSSA^t^	CSB stabilization Transcription‐coupled nucleotide excision repair (TC‐NER)	(1, 44, 45)
DNMT1^u^	Replication Cell proliferation and migration	(1, 4, 56)
PTEN	Tumor metastasis Cell apoptosis Tumor suppression	(1, 57, 58)
Claspin	Tumor suppression	(43)
FOXO^v^		(16)

a IκB kinase γ.

b Tumor necrosis factor (TNF) receptor associated factor 6.

c Latency‐associated nuclear antigen.

d Viral interferon regulatory factor.

e RNA transcriptional activator.

f Epstein‐Barr nuclear antigen 1.

g Forkhead box P3.

h Coiled-coil domain containing 6.

i Checkpoint kinase 1.

j Xeroderma pigmentosum complementation group C.

k Cockayne syndrome B.

l Helicase-like transcription factor.

m Checkpoint protein with FHA and RING domains.

n Ubiquitin‐like, containing PHD and RING finger domains 1.

° Mediator of DNA damage checkpoint 1.

p Plant homeodomain finger‐containing protein 8.

q Annexin‐1.

r Ring finger protein 168.

s Ring finger protein 169.

t UV-stimulated scaffold protein A.

u DNA methyltransferases 1.

v Forkhead box O.

This review summarizes various functions of USP7 in diverse cellular processes and cancers, as well as the regulatory mechanisms of its expression levels and activity, aiming to emphasize the necessity of research on USP7, and provide a better understanding of USP7-related biological processes and cancer.

## 2 Physiological and Pathological Roles of USP7

USP7 has been shown to be involved in a variety of biological processes in cells, and is associated with many cancers through the regulation of its downstream substrates.

### 2.1 USP7 and Immune Signaling

Multiple proteins have been shown to be the substrates of USP7 in immune signaling. USP7 may promote inflammatory signaling by stabilizing NF-κB (19). IκB kinase γ (IKK-γ) polyubiquitination and IκB degradation can be suppressed when USP7 interacts with HSCARG, which subsequently inhibits NF-κB signaling (20). In the innate immune response, USP7 promotes deubiquitination of TRAF3 and TRAF6 by binding to vIRF2, resulting in extended transactivation of TRAF3 and TRAF6, thereby regulating the interferon response (21). Moreover, USP7 reduces NFκB -mediated innate immune responses through TRAF6 and IKK-γ deubiquitination, which appears to be directed by the USP7-ICP0 complex (22, 23). This may be the mechanism by which USP7 prevents HSV from innate host immunity (22).

During the adaptive immune response, USP7 contributes to the evasion of tumors by promoting the deubiquitination and stability of proteins, such as Tip60 and Forkhead box P3 (Foxp3) (31), which act a pivotal part in promoting the suppressive functions of both Treg and Teff (59). In mice treated by DSS, it appears that a novel small molecule cambogin relieves the symptoms of enteritis and reduces the production of pro-inflammatory cytokines (60). This may be due to the effect of cambogin on the promoter of USP7, which promotes the deubiquitination and expression of Foxp3, and reduces inflammation by affecting the immune response mediated by Treg cells (61). DNMT1 and Ubiquitin‐like, containing PHD and RING finger domains 1 (UHRF1) play essential roles in modulating Treg function and development (62), and the stability of the former is also regulated by USP7 (62). In mouse models, USP7 deletion in Treg cells has been found to induce lethal systemic autoimmunity (63). In addition, deleting USP7 also down-regulates the expression of many transcription factors that are essential to Treg cell development (48), and is correlated to the increased-level of IFNγ and IL-2 expression (62), resulting in the impairment of Treg suppressive functions (31), which seems to promote antitumor immunity (62) and break immunotolerance (31). Therefore, given the suppressive functions of USP7 inhibitors in regulating Foxp3+Treg cells, USP7 could be a potential target in cancer immunotherapy.

### 2.2 DNA Damage Responses

**Figure 2:**
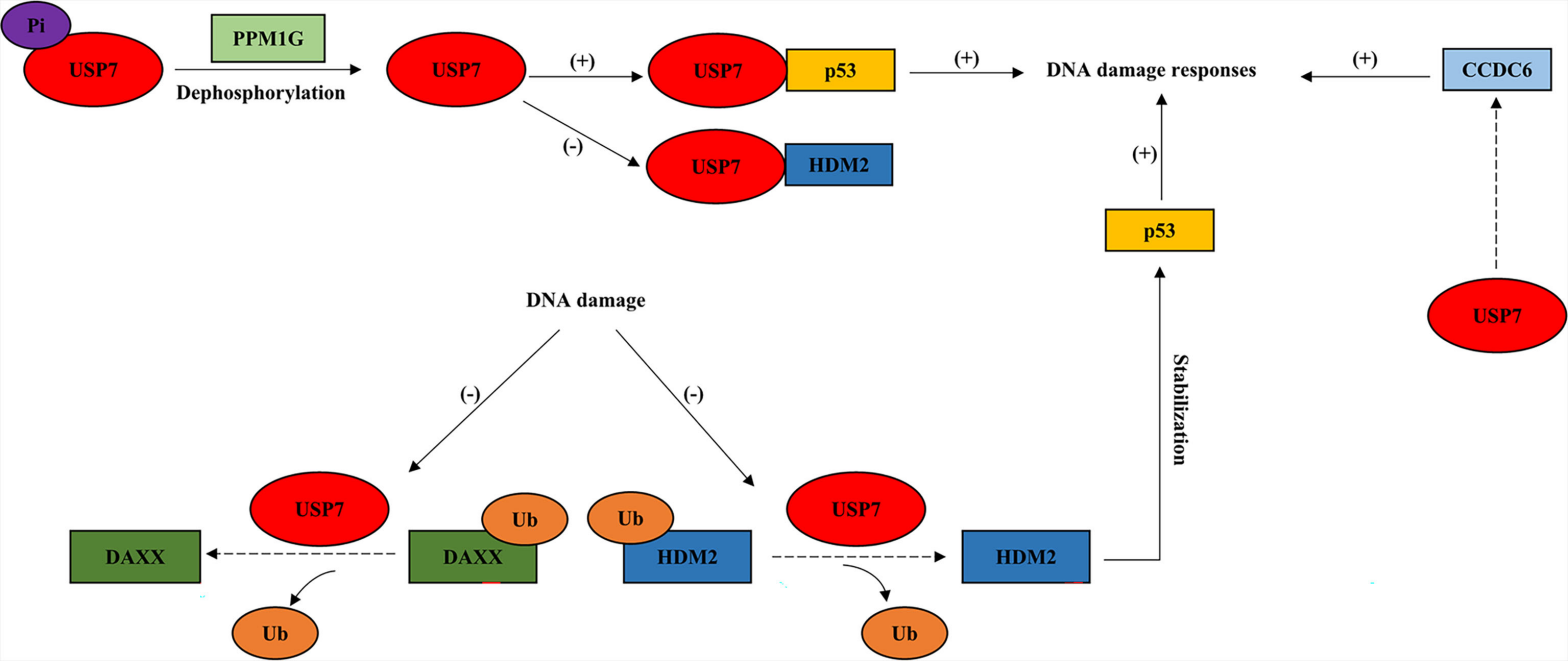
USP7 interacts with tumor suppressors in response to DNA damage. This model describes how USP7 effects DNA damage responses by interacting with tumor suppressive proteins, p53 and CCDC6. Dotted arrow: deubiquitination; Brackets with a “+”: promotional effect; Brackets with a “-”: inhibitory effect. USP7, ubiquitin-specific peptidase 7; DAXX, death-associated protein 6; PPM1G, protein phosphatase 1G; CCDC6, coiled-coil domain containing 6.

**Figure 3:**
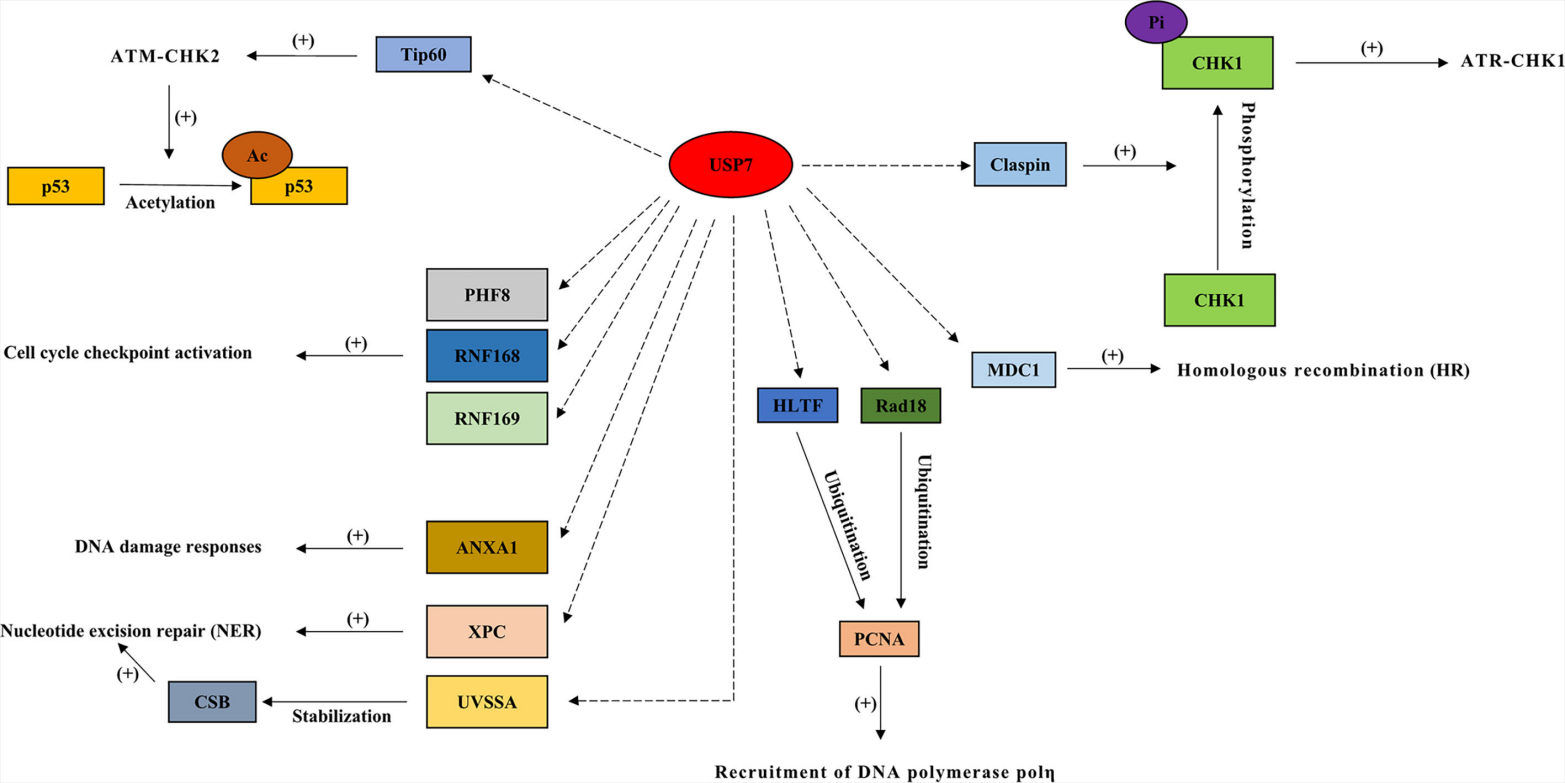
Interactions between USP7 and its substrates in double-strand DNA breaks, homologous recombination repair (HRR), cell cycle checkpoint activation, nucleotide excision repair (NER), and DNA damage bypass. In response to DNA damage, USP7 regulates multiple proteins in double-strand DNA breaks (ATR-CHK1 and ATM-CHK2 signaling cascades), homologous recombination repair (HRR), cell cycle checkpoint activation, nucleotide excision repair (NER), and DNA damage bypass. Dotted arrow: deubiquitination; Brackets with a “+”: promotional effect; Brackets with a “-”: inhibitory effect. USP7, ubiquitin-specific peptidase 7; CHK1, checkpoint kinase 1; MDC1, mediator of DNA damage checkpoint 1; PHF8, plant homeodomain finger‐containing protein 8; RNF168, ring finger protein 168; RNF169, ring finger protein 169; XPC, xeroderma pigmentosum complementation group C; UVSSA, UV-stimulated scaffold protein A; HLTF, helicase-like transcription factor.

In response to DNA damage, USP7 regulates multiple proteins in double-strand DNA breaks (ATR-CHK1 and ATM-CHK2 signaling cascade), homologous recombination repair (HRR), cell cycle checkpoint activation, nucleotide excision repair (NER), and DNA damage bypass, where p53 functions as one of the key factors (**Figures 2** and **3**).

During DDR, USP7 acts as a pivotal role in the regulation of genomic stress and cell fate through deubiquitinating and stabilizing the key factor, tumor suppressor p53 (**Figure 2**). The interaction between p53 and USP7 leads p53 to effect DNA damage repair at the level of gene transcription (35). DNA damage results in USP7 dephosphorylation *via* protein phosphatase 1G (PPM1G), which is in a way dependent on ATM (64). This causes lower USP7 affinity to HDM2 and increases its affinity towards p53 instead, driving p53-dependent DDR (8, 64). DNA damage also leads to the disrupted association between USP7 and DAXX, resulting in the ubiquitination of HDM2 and stabilization of p53 later (21). Moreover, USP7 is also required for DDR and DNA repair *via* its interaction with another tumor suppressor, coiled-coil domain containing 6 (CCDC6) (1), which has been shown to produce the pro‐apoptotic protein (65) (**Figure 2**).

During double-strand DNA breaks (DSBs), USP7 acts as an essential part of the ATR-CHK1 branch (8) by regulating checkpoint kinase 1 (CHK1), thereby playing a vital role in DDR (41). USP7 promotes CHK1 phosphorylation mediated by ATR (1), and is required for the regulation of claspin that is key to CHK1 activation (66). Another crucial part of DDR, the ATM-CHK2 pathway, is also regulated by USP7 (67). USP7 indirectly activates p53 through deubiquitinating Tip60 (21), which activates the ATM-CHK2 signaling cascade through acetylating ATM, causing the acetylation and activation of p53 (33).

USP7 plays a role in recruiting DNA repair proteins towards DSB lesions, and influences homologous recombination (HR) through regulating the mediator of DNA damage checkpoint 1 (MDC1) (51). In cells depleted of MDC1, the failure of DNA repair proteins recruitment is found after DNA damage, and a similar defect is found in USP7-depleted cells (51). In chronic lymphocytic leukemia (CLL), inhibition of USP7 suppresses HRR and induces cell death in a manner independent of ATM-p53 (1). Additionally, E3 ligase RNF168 deubiquitinates the histones that function as a signal in the recruitment of DNA damage factors (21), and controls the access of proteins related to DDR to chromatin (11, 12). USP7 has been shown to regulate cell cycle checkpoint activation, in response to DNA damage, through the deubiquitination and stabilization of RNF168, RNF169, and the histone demethylases, plant homeodomain finger‐containing protein 8 (PHF8) (4, 8, 11, 12).

Protein XPC plays a vital part in recognizing damage, functions as a lesion sensing factor (10), and initiates NER by interacting with USP7 following DNA damage induced by UV (1, 10). ANXA1 (Annexin‐1), one of the substrates of USP7, acts as a stress protein or protective protein during the UV‐induced DDR (53). Additionally, UVSSA (UV-stimulated scaffold protein A) functions as an initiating factor of transcription‐coupled nucleotide excision repair (TC-NER) (44). During the UV-induced TC-NER process, USP7 interacts with RNA polymerase II and UVSSA to stabilize CSB (cockyane syndrome B) that also plays a vital role in the regulation of TC-NER, in response to DNA damage (1). Studies have shown that USP7 depletion causes TC-NER deficiency, such as reduced UV survival and decreased recovery in RNA synthesis following UV (44, 45).

USP7 regulates the stability of trans-lesion synthesis (TLS) DNA polymerase, polη, *via* a USP7‐HLTF‐PCNA molecular network, in response to DNA damage (54, 55). Following genotoxic stress, USP7 is involved in the regulation of error-free bypass replication through the interaction with helicase-like transcription factor (HLTF), and helps elongate nascent daughter strand DNA through Rad18 (46) (68). The defect in elongation of nascent DNA strands is found in cells depleted of USP7 after UV exposure, which may be due to the regulation of USP7 on PCNA sliding clamp ubiquitination (46, 55, 69, 70). In addition, USP7 also plays a role in DNA damage tolerance by regulating Rad18 stability (8). Lower levels of HLTF and Rad18, suppressed PCNA ubiquitination, and suppressed Polη foci formation are all found in cells depleted of USP7 (35).

### 2.3 USP7 and Cancers

Accumulated evidence has shown that abnormal expression and activity of USP7 are associated with a variety of cancers. Here is a summary of the mechanisms of USP7 for effecting tumor initiation and progression through interaction with its downstream proteins, as detailed below.

#### 2.3.1 Cell Proliferation

USP7 regulates MDM2/MDMX-p53 circuitry and controls the stabilities of related proteins, thereby influencing cell proliferation, cancer initiation, and progression (31). In prostate cancer, inhibition of USP7 expression is found to have an anti-proliferative effect on cancer cells (71). USP7 also influences the cell cycle by stabilizing the Ki-67 antigen (49), which has been shown to help maintain mitosis and heterochromatin (72). In addition, USP7 regulates the target of the cell cycle, cyclin A2, through the deubiquitination and stabilization of PHF8 demethylase, which acts as an up-regulator of cyclin A2 (48, 52). Moreover, upregulation of USP7, PHF8, and cyclin A2 has also been found in breast cancers, colon and rectum cancers (48).

#### 2.3.2 Cell Migration

In colorectal cancer (CRC) (50) and medulloblastoma (73), USP7 overexpression contributes to the increased rate of cell proliferation and migration through its association with histone LSD1 and DNMT1 (4). In prostate cancer, emerging evidence has proved that USP7 contributes to tumor migration and invasion through stabilizing EZH2 (74). The decreased rate of cell migration and invasion is found in PC3 and DU145 depleted of USP7 (74). Combined treatment with USP7 and EZH2 inhibitors has been shown to reduce the migration and invasion of cancer cells (74). In osteosarcoma (OS), overexpression of USP7 significantly improves the ability of cell migration and invasion, while USP7 depletion has the opposite effects, indicating the key role of USP7 in regulating the migratory and invasive ability of osteosarcoma cells (75).

#### 2.3.3 Cell Metastasis

USP7 is involved in tumor metastasis through deubiquitination and localization of PTEN, which results in the inactivation of PTEN (57, 58). It has been shown that USP7 promotes epithelial-mesenchymal transition (EMT) of OS cells through Wnt/β-catenin signaling (75), which is one of the key parts in promoting cell metastasis (76). In non-small cell lung cancers (NSCLC), USP7 dysregulation is also related to EMT and cell metastasis, leading to poor prognosis (77). Moreover, in both epithelial ovarian cancer (78) and prostate cancer (31), the overexpression of USP7 is found to promote cell invasion, the increased expression level of which is also related to poor survival of ovarian cancer patients (78).

#### 2.3.4 Cell Immunosuppression

During an innate immune response, USP7 has been found to diminish NFκB through its interaction with ICP0 (22, 23). ICP0-USP7 complex participates in the deubiquitination and inactivation of both IKK-γ and TRAF6 (22), leading to the inhibition of innate immunity. Besides, IKK-γ polyubiquitination and IκB degradation can be suppressed when USP7 interacts with HSCARG (20, 21), which subsequently inhibits NF-κB signaling (20). It has also been shown that the recruitment of USP7 by HSV-1 results in HSV-1 evasion of innate immune response (79). Moreover, recent studies have identified the role of USP7 as a target of VP24 from the Ebola virus, which is a protein that plays a pivotal part in innate immune evasion (32).

In the stage of the adaptive immune response, USP7 promotes tumor immune escape through deubiquitination and stabilization of both Foxp3 and Tip60 (31), which play key roles in inhibiting Treg and Teff (59, 63). Conversely, USP7 knockdown results in the instability of Foxp3, which subsequently impairs the immunosuppressive function of Treg (62, 80), and promotes tumor suppression mediated by the immune system (63). In addition, induced lethal systemic autoimmunity has been found in mouse model with USP7 depletion (63). Taken together, all of these data indicate the essential role of USP7 in modulating the immune response.

#### 2.3.5 Cell Apoptosis

**Figure 4:**
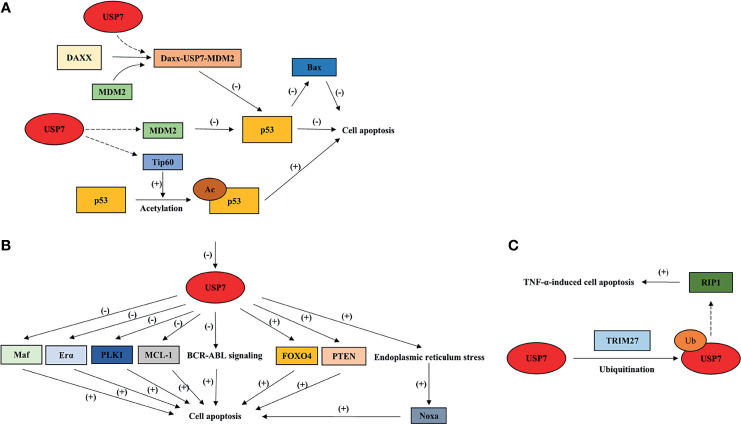
Overview of USP7 effects on cell apoptosis by interacting with its substrates. This model summarizes how USP7 effects p53-dependent cell apoptosis **(A)** and p53-independent cell apoptosis **(B, C)**. Dotted arrow: deubiquitination; Brackets with a “+”: promotional effect; Brackets with a “-”: inhibitory effect. USP7, ubiquitin-specific peptidase 7; FOXO4, forkhead box O4; TRIM27, tripartite motif protein 27; TNF-α, tumor necrosis factor-alpha.

Studies have demonstrated the duality of USP7 functions in cancer progression. In other words, USP7 displays tumor-promoting and/or -suppressive functions under different conditions (**Figure 4**).

USP7 effects p53-dependent apoptosis through its interaction with p53 and negative regulator of p53, MDM2. In most cases, USP7 shows a higher affinity to MDM2, leading to p53 ubiquitination, which indicates the tumor-promoting effect of USP7. Daxx plays an anti-apoptotic function in cancer cells through the formation of ternary complex USP7-MDM2-Daxx, which stabilizes MDM2, and later promotes degradation of p53 (5). In NSCLC, defect of USP7 leads to p53 upregulation, subsequently causing p53-induced apoptosis through p53 downstream target, Bax (81). In colorectal cancer cell lines, it has been revealed that FAM188B may be the mediator that controls the combination between USP7 and p53/MDM2 (82). The interaction between USP7 and FAM188B effects p53 stability, which may be one of the reasons why p53-dependent apoptosis is induced by FAM188B downregulation (82). Notably, USP7 also induces p53-dependent apoptosis by interacting with Tip60, which is involved in K120 acetylation that is located in the DNA-binding domain of p53 (35).

Accumulated studies have also shown the apoptotic function of USP7 inhibition in a p53-independent manner. In chronic myelogenous leukemia (CML), USP7 is required for stabilization of BCR-ABL and activation of BCR-ABL signaling (83).Inhibiting USP7 was shown to cause BCR-ABL destabilization and to trigger apoptotic signaling pathways (83). In CLL cells, USP7 inhibition induces cell apoptosis by restoring nuclear localization of PTEN (84). Additionally, FOXO4 transcriptional activity is promoted through USP7 inhibition, which induces apoptosis in an HDM2-independent manner (79). In multiple myeloma cell (MM) cells, Maf transcriptional activity is promoted by USP7 through deubiquitination and stabilization of Maf (85). Inhibiting USP7 leads to Maf downregulation, thereby inducing apoptosis (85). USP7 also promotes apoptotic escape of breast cancer cells by deubiquitinating and stabilizing Erα (86). Silencing or inhibiting USP7 induces apoptosis and inhibits cell growth in breast cancer (86). Moreover, USP7 inhibition may cause aberrant mitosis and apoptosis through downregulation and degradation of PLK1 (87). Studies have also shown that USP7 participates in the stabilization of anti-apoptotic protein MCL-1, leading to the upregulation of MCL-1 levels in tumor cells (88). In addition, inhibiting USP7 induces endoplasmic reticulum stress triggered by ubiquitinated proteins accumulation, which promotes NOXA expression levels and leads to NOXA-induced apoptosis (89). Interestingly, USP7 also promotes TNF-induced apoptosis through its combination with TRIM27 and receptor-interacting protein 1 (RIP1) (90), indicating the tumor-suppressive role of USP7.

Collectively, USP7 plays a complex role in regulating apoptosis. Specific mechanisms that could explain how USP7 interacts with its substrates in apoptosis are needed to explore the different roles of USP7 in various cancers.

## 3 Regulation of USP7 Activity and Expression Levels

With multiple downstream target proteins, USP7 is also influenced by a plethora of regulators. Here is a review of the mechanisms of USP regulation, including the regulation of its expression levels and deubiquitinase activity.

### 3.1 Transcription Regulation

Several transcription factors are involved in the regulation of USP7 (**Table 2**). USP7 ubiquitination is found to be catalyzed by ICP0, a protein derived from a virus that functions as an E3 (96), leading to USP7 degradation and decreased levels (95). Downregulation of USP7 mediated by signal transducer and activator of transcription 3 (STAT3) has been found in colon cancer (94). Forkhead box O6 (FOXO6) upregulates USP7 transcription by binding to its promoter (91). In lung carcinoma, cell proliferation is found to be inhibited through the promoted expression of USP7 induced by FOXO6 (91). In acute lymphoblastic leukemia, NOTCH1 contributes to the upregulation of USP7 levels, and USP7, in turn, influences the stability of NOTCH1, which possibly forms a positive-feedback interaction between the two proteins (92). And the similar feedback loop has been demonstrated between USP7 and PHF8 (48, 93). Some regulators of USP7 can indirectly influence its downstream substrates, and thus regulate related processes. For instance, PPM1G reverses USP7’s functions on MDM2 and p53 following the down-regulation of USP7 caused by PPM1G (64). Moreover, LKB1 activation induces HuR to translocate from the nucleus to the cytoplasm, leading to stabilization of USP7 mRNAs and phosphorylation of p53, thereby influencing apoptosis (97). Remarkably, the expression of USP7 is also found to be enhanced by the HIV-1 virus, because USP7 plays an essential part in regulating the stability of the trans-activator of transcription (TAT) (21), which is involved in regulating the levels of HIV genes (98).

**Table 2:** Regulators of USP7 levels.

Regulators		References
Up-regulators	FOXO6^a^	(91)
	NOTCH1	(92)
	PHF8^b^	(48, 93)
Down-regulators	STAT3^c^	(94)
	PPM1G^d^	(64)
	ICP0	(95)

a Forkhead box O6.

b Plant homeodomain finger‐containing protein 8.

c Signal transducer and activator of transcription 3.

d Protein phosphatase 1G.

### 3.2 Posttranslational Modifications

USP7 activity is regulated through PTMs, mainly including ubiquitination and phosphorylation, as detailed below (**Table 3**).

**Table 3:** Regulators of USP7 activity.

Regulators	Effects	References
ICP0	Ubiquitination	(95)
Trip12		(99)
TRIM27		(90)
CK2^a^	Phosphorylation	(64)
BCR-ABL		(100)
PPM1G	Dephosphorylation	(64)

a Casein kinase 2.

#### 3.2.1 Ubiquitination

The region where USP7 is ubiquitinated is located near the site where it binds to the E3 ubiquitin ligase ICP0 (96). ICP0 functions as a catalyzer in USP7 ubiquitination, leading to its degradation and decreased levels (95). The feedback interaction is found between USP7 and ICP0, as USP7 could in turn participate in the deubiquitination of ICP0 (95). Interestingly, the ubiquitination of USP7 is also found in cells that do not express ICP0 (5), indicating that USP7 could be ubiquitinated in an ICP0-independent way. However, the specific DUB and E3 ligases that participate in the ubiquitination of USP7 have not been identified (5). Recently, another E3 ligase Trip12 has also been shown to ubiquitinate USP7 (99). In addition, polyubiquitinated USP7 promotes TNF-α induced apoptosis by interacting with E3 ligase TRIM27 on Lys869 and RIP1 (90). However, USP7 protease activity does not always require ubiquitination, without which USP7 is still able to deubiquitinate MDM2 and p53 (5).

#### 3.2.2 Phosphorylation

Casein kinase 2 (CK2) improves the affinity of USP7 or USP7 isoform (USP7S) to HDM2 or MDM2 by phosphorylating USP7 or USP7S at Ser18, while dephosphorylating USP7 or USP7S enhances their affinity for p53 (64). In addition, the effect of USP7/USP7S on HDM2 and p53 could be reversed by the ATM-dependent phosphatase PPM1G (64). Activated ATM signaling promotes USP7/USP7S dephosphorylation mediated by PPM1G, inactivates and downregulates USP7/USP7S, and leads to decreased levels of MDM2 and p53 upregulation (64). Moreover, in chronic leukemia, it has been shown that BCR-ABL could aberrantly phosphorylate USP7 at Tyr243, contributing to the function of USP7 as a deubiquitinase for PTEN (8, 100). And dysregulated PTEN caused by phosphorylated USP7 is associated with the pathogenesis of leukemia (100).

## 4 Conclusion

This review summarizes the physiological and pathological functions of USP7 in immune signaling, DNA damage response, and cancers, as well as the regulatory mechanisms of its expression and activity. However, in respect of the various functions of USP7 in cell processes, the association between USP7 and cancers, and USP7 targeted cancer therapy, there are still many fields that need to be further explored.

In order to illuminate the real roles of USP7 in carcinogenesis, it is requisite to further investigate the dual effects of USP7 in tumor regulation. Additionally, considering the critical part that tumor suppressor p53 plays in various cancers, targeting the USP7-MDM2-p53 axis will still be one of the popular topics in the future. Further research is needed to explore whether there are other regulatory factors of USP7 on p53 and MDM2, and USP7-specific transcription inhibitors and factors, the discovery of which could have future implications for the study of cancer with an intact p53 regulatory axis.

Accumulated studies have shown p53 mutation and p53-independent role of USP7 in quite a few cancer cases. For example, in melanoma cells, USP7 expression levels are found to increase in a p53-independent manner (101), and inhibiting USP7 results in endoplasmic reticulum stress and DNA damage, which is in a way independent of p53 (102, 103). Most USP7 inhibitors are developed based on wild type (WT) p53, however, a recent study has shown that inhibiting USP7 plays a suppressive role in tumor progression with both WT p53 and p53 mutation (104). All of these indicate that USP7 may effect carcinogenesis by interacting with other substrates, which needs to be further explored.

Given the association between USP7 and a few signaling pathways in tumor progression [such as NF-κB pathway (105), Wnt/β-Catenin pathway (106), Hippo pathway (107), and NOTCH pathway (108)], more related mechanistic investigations on USP7 regulation are needed to develop new targeting strategies in cancer therapy.

In addition to the nuclear deubiquitinase role, USP7 also participates in the recycling of endosomal proteins (109). Exploring additional functions of USP7 is required for improving our understanding of its roles in biological and pathological processes.

In the process of developing USP7 inhibitors, there are still some issues that remain to be solved. Theoretically, for instance, the full-length structure of USP7 has not been fully studied, and co-crystal structures of USP7 in complex with small molecules also remain unclear. How USP7 is activated, how does USP7 recognize its various substrates, and how USP7 inhibitors work? A complete and deeper understanding of these fundamentals of molecular theory and mechanisms are essential to the development of USP7 inhibitors. In general, many reported USP7 inhibitors so far have been shown to have poor selectivity, low specificity, and low efficacy (low micromolar potency). Unsatisfactory pharmacokinetic characteristics of USP7 inhibitors also limit their evaluation *in vivo*. In order to improve their selectivity and specificity, manipulation of USP7’s affinity to specific target substrates may be feasible, which requires support on the theoretical basis of USP7 recognition mechanisms. Further studies should also focus on the development of better screening methods, which will undoubtedly improve the accuracy and efficiency of inhibitor evaluation. Additionally, it appears that quite a few USP7 inhibitors could also target other related DUBs (such as USP47, which has high homology with USP7) with similar potency. As a result, this kind of cross effect adds difficulty in determining the contribution of USP7 inhibition to the effect of the tested compound in cancer progression.

According to the discussion above, the development of USP7 inhibitors is still in a primary and long-term stage. However, notably, there have also been some breakthroughs in recent years. The inhibition mechanism of P22077 and P50429 on USP7 has been revealed by Pozhidaeva et al. (110). Gavory et al. have reported the first reversible USP7 inhibitor with high efficacy (nanomolar potency) and high selectivity, which has been shown to have no interaction with USP47 when targeting USP7 (111). Schauer et al. reported the first irreversible USP7 inhibitor with high efficacy (sub-nanomolar potency) and high selectivity (112). Moreover, P5091 has been shown to have efficacy and very low toxicity in tumor treatment *in vivo* (113–115). In sum, all of the issues are not insurmountable for the development of more ideal inhibitors.

Excitingly, the combination of USP7 inhibitors and several current major cancer treatments has been shown to possess promising therapeutic prospects, mainly including chemotherapy, radiotherapy, and immunotherapy (such as PD-L1 treatment), which will be one of the study directions in the future. For example, the resistance to bortezomib appears to be overcome through the combination of USP7 inhibitors P22077 and cytotoxic drugs used for chemotherapy (4, 116). USP7 inhibition may also improve the effectiveness of Olaparib treatment by increasing drug sensitivity through CCDC6 downregulation (117, 118). In addition, given the essential roles of PHF8 and CHK1 that act as USP7 substrates in DNA damage response, combination of USP7 inhibitors and chemotherapy/radiotherapy may reduce breast cancer cell resistance (42, 63), which is expected to accelerate the development of breast cancer therapy. Besides, targeting USP7 inhibits cell apoptosis in response to chemo-/radiotherapy (5), indicating the promising role of USP7 inhibitors in reducing adverse reactions in cancer treatment. Moreover, during anti-tumor immunotherapy, it has been shown that USP7 inhibition causes PD-L1 increased level (119) and IL-10 downregulation by attenuating Treg function (120), which may contribute to enhancing the therapeutic effect of PD-L1 treatment. Meanwhile, given the suppressive function of USP7 inhibitors in regulating Treg cells (31), a non-negligible concern about the safety of inhibitors is raised: Whether the host immune response will be impaired due to functional inhibition of Treg? Therefore, in order to ensure the availability and effectiveness of USP7 inhibitors in cancer therapy, evaluation of drug safety is very necessary, which requires more data from *in vivo* experiments.

In sum, clinical application of targeting USP7 possesses a broad prospect, and research on USP7 is necessary, which still has a long way to go.

## Author Contributions

Conceptualization, TO. Writing—original draft preparation, LZ and TO. Writing— review and editing, LZ, TO, ML, and TH. Visualization, ML, TH, AMHS, and WM. Supervision, AMHS, WM, and NZ. All authors contributed to the article and approved the submitted version.

## Funding

The present study was supported by the National Natural Science Foundation of China (grant no. 81760447(TO); grant no. 81960247(NZ), Project of Science and Technology Department of Jiangxi Province (grant no. S2019QNJJB1056) (NZ), grant no. 20202BABL206099 (WM) and Jiangxi Provincial Education Department Project [grant no.GJJ180054 (TO); grant no. GJJ180116(NZ)].

## Conflict of Interest

The authors declare that the research was conducted in the absence of any commercial or financial relationships that could be construed as a potential conflict of interest.

## Publisher’s Note

All claims expressed in this article are solely those of the authors and do not necessarily represent those of their affiliated organizations, or those of the publisher, the editors and the reviewers. Any product that may be evaluated in this article, or claim that may be made by its manufacturer, is not guaranteed or endorsed by the publisher.
